# Electroencephalogram Alpha Oscillations in Stroke Recovery: Insights into Neural Mechanisms from Combined Transcranial Direct Current Stimulation and Mirror Therapy in Relation to Activities of Daily Life

**DOI:** 10.3390/bioengineering11070717

**Published:** 2024-07-15

**Authors:** Chia-Lun Liu, Ya-Wen Tu, Ming-Wei Li, Ku-Chou Chang, Chih-Hung Chang, Chih-Kuang Chen, Ching-Yi Wu

**Affiliations:** 1 Department of Occupational Therapy, Chang Gung University, Taoyuan 33302, Taiwan; d000019299@cgu.edu.tw; 2 Department of Physical Medicine and Rehabilitation, Sijhih Cathay General Hospital, New Taipei 221, Taiwan; yawentu@gmail.com (Y.-W.T.); otdefi@gmail.com (M.-W.L.); 3 Division of Cerebrovascular Diseases, Department of Neurology, Kaohsiung Chang Gung Memorial Hospital, Kaohsiung 80756, Taiwan; kcchang@adm.cgmh.org.tw; 4 Long-Term Care Service Center, Kaohsiung Chang Gung Memorial Hospital, Kaohsiung 80756, Taiwan; 5 Department of Medicine, Chang Gung University College of Medicine, Taoyuan 33302, Taiwan; albert.ckchen@gmail.com; 6 Program in Occupational Therapy, Washington University School of Medicine, St. Louis, MO 63108, USA; chih-hung.chang@wustl.edu; 7 Department of Medicine, Washington University School of Medicine, St. Louis, MO 63110, USA; 8 Department of Orthopaedic Surgery, Washington University School of Medicine, St. Louis, MO 63130, USA; 9 Department of Physical Medicine and Rehabilitation, Chang Gung Memorial Hospital at Linkou, Taoyuan 33302, Taiwan; 10 Healthy Aging Research Center, Chang Gung University, Taoyuan 333, Taiwan

**Keywords:** stroke, activities of daily function, EEG oscillatory activity, neurorehabilitation, tDCS

## Abstract

The goal of stroke rehabilitation is to establish a robust protocol for patients to live independently in community. Firstly, we examined the impact of 3 hybridized transcranial direct current stimulation (tDCS)-mirror therapy interventions on activities of daily life (ADL) in stroke patients. Secondly, we explored the underlying therapeutic mechanisms with theory-driven electroencephalography (EEG) indexes in the alpha band. This was achieved by identifying the unique contributions of alpha power in motor production to ADL in relation to the premotor cortex (PMC), primary cortex (M1), and Sham tDCS with mirror therapy. The results showed that, although post-intervention ADL improvement was comparable among the three tDCS groups, one of the EEG indexes differentiated the interventions. Neural-behavioral correlation analyses revealed that different types of ADL improvements consistently corresponded with alpha power in the temporal lobe exclusively in the PMC tDCS group (all *r*s > 0.39). By contrast, alterations in alpha power in the central-frontal region were found to vary, with ADL primarily in the M1 tDCS group (*r* = −0.6 or 0.7), with the benefit depending on the complexity of the ADL. In conclusion, this research suggested two potential therapeutic mechanisms and demonstrated the additive benefits of introducing theory-driven neural indexes in explaining ADL.

## 1. Introduction

Stroke is the second leading cause of mortality and the third leading cause of long-term disability worldwide [1]. While current rehabilitation methods have made noticeable advances in improving motor impairment, research still struggles to establish a robust protocol for improving activities of daily living (ADL), which is the ultimate goal of a rehabilitation program. Therefore, it is essential to continue exploring new approaches. Upper extremity function is commonly involved in most daily activities, making it the primary focus of most neurorehabilitation methods. Equally important, in addition to strength enhancement, recovery in ADL or even instrumental activities of daily living (IADL) rely more on cognitive functions such as motor planning. These functions can be challenging to detect through behavioral measurements or assessments. An electroencephalogram (EEG) has good temporal resolution, and its relevant neural indexes have been documented to link different aspects of motor function [2,3]. However, the neurophysiological indexes associated with ADL are still unclear. To search for a generalized neural index for tracking treatment effects on ADL, we decided to leverage a theoretical framework to explore its potential neural indexes.

One of the promising approaches to enhancing functions related to ADL is non-invasive brain stimulation [4,5]. Transcranial direct current stimulation (tDCS) is a type of non-invasive brain stimulation that involves applying low-intensity electrical current directly to the scalp. Beside the EEG mentioned earlier, functional near-infrared spectroscopy has also been employed to examine the impact of tDCS stimulation, particularly investigating if the neural response corresponds to the hemodynamic response measured by the functional near-infrared spectroscopy [6]. This clearly implies the effectiveness of tDCS in stimulating the targeted brain region. Hypothetically, this technique makes tDCS a potentially useful tool for modulating neuronal inhibitory and excitatory networks in both the affected and non-affected hemispheres post-stroke to enhance recovery.

Additionally, recent evidence suggests that a combined rehabilitation approach could further enhance treatment effects [4]. For instance, a combination of therapeutic robots and occupational therapy has been proven effective in improving ADL [5]. Similarly, a recent study integrating constraint-induced movement therapy and tDCS found improvements in ADL that lasted up to three months [4]. While these encouraging results are mainly found in acute stroke cases, evidence for chronic stroke patients remains weak due to variations in study design and tDCS protocols [7]. Although the effectiveness of combined tDCS treatments has continued to advance, the rationale for combining tDCS tends to be exploratory or based on the assumption that the effects are additive. Additionally, the underlying therapeutic mechanisms and relevant neural indexes remain largely unknown. We proposed that a gating-by-inhibition model [8], along with its corresponding EEG indexes, could serve as an ideal model. These indexes could be used to establish a therapeutic mechanism for hybrid tDCS by combining tDCS and mirror therapy (MT). Guided by this conceptual model, modifications to the protocol can be pursued more effectively.

It is well established that motor-related regions play a critical role in producing movement. Other regions, such as the temporal lobe, are also involved in movement. Building on this, the gating-by-inhibition model [8] proposed that at the neurophysiological level, the execution of an action is accompanied by a distinct evolving spatial pattern of EEG activity, particularly within the alpha frequency band. Prior to movement initiation, there is an increase in oscillatory activity in the alpha band in the temporal region, followed by a further increase during movement execution [8]. The rise in alpha power in the temporal area has been interpreted to reflect the inhibition of cortical activity. The functional roles of this suppressed cortical activity have been linked to cognitive interference during motor preparation [9] and motor monitoring during movements [10]. Previous findings also indicate that greater alpha power (i.e., stronger inhibition) in the left temporal region varies with expertise [11], performance [12], and practice [13]. These studies demonstrate the potential of alpha power as an index of neural plasticity.

During the rehabilitation process, neuroplasticity can be achieved by circuit rewiring through strengthening synaptic connections with long-term potentiation and weakening connections with long-term depression [14]. This emphasizes a relearning process that directly relates to the rearrangement of the motor network. From a neurophysiological perspective, as previously mentioned, certain spatiotemporal contributions (such as from the temporal lobe) may not be helpful for circuit rewiring; inhibiting the functional role associated with this region could instead promote the efficiency of motor generation. In other words, reducing unnecessary cognitive interference during movement could shield motor efficiency from motor-related interference. The goal of a rehabilitation program is to restore the patient’s functional ability for daily life. The extent to which such shielding of motor efficiency contributes to different aspects of recovery in rehabilitation remains an unexplored issue.

As the hybrid approach did not consistently show greater gains than neurorehabilitation alone, it suggests that some parameters of tDCS still need to be optimized [15]. The most prominent factor is the tDCS application site. The primary motor cortex (M1) is usually targeted for motor recovery. Since the M1 has direct projections to spinal motoneurons, it has long been considered a primary hub for motor control and execution. Its role in motor learning [16] and motor sequence learning [17] has also been established. A recent neuroimaging study found the M1 to be a hub of the motor network, interacting with other cognitive networks for motor learning [18]. Generally, it appears that the M1 is mainly involved in motor-related functions during rehabilitation. Compared to the M1, the premotor cortex (PMC) is situated upstream in the motor network and is known for its role in various motor-related functions, such as motor learning and cognition [19,20]. Activation of the PMC is observed during the early phase of motor learning [19], and focused activation of the ipsilesional PMC is especially important for efficient learning in patients after stroke [21]. When considering the application site with combined therapy, the PMC is part of the mirror neuron system and has been suggested to be associated with MT [22]. During MT, activation of the bilateral PMC is also found to couple with the activation of the M1 [23]. This implies that combining PMC tDCS and MT may guide plastic processes toward more meaningful motor and functional improvement, particularly for ADL.

Both the PMC and M1 have been suggested as promising sites for tDCS application; however, they may rely on different paths in ADL recovery. It can be tentatively speculated that pure motor function in ADL recovering is mainly associated with the M1, while motor relearning involving cognition (motor planning, motor sequence, communicating) is more strongly associated with the PMC. According to the gating-by-inhibition model, it is speculated that temporal alpha activity, which relates to the inhibition of non-motor interference, corresponds to the PMC, while alpha activity in the central-frontal area is associated with the M1.

The first objective of this study was to assess the impact of combined tDCS and MT on ADL measures and EEG indexes. Compared to behavioral measures, EEG indexes provide more temporal information, allowing for the identification of their functional contributions to motor function. Consequently, individual variations in EEG measures could be meaningful and used to explain clinical improvements, such as those in ADL. Additionally, tDCS is not only intended to augment the treatment effect but also to amplify the functional involvement in motor execution if it affects the relevant functional processes. To the best of our knowledge, no study has directly tested these relationships. To obtain a reliable estimation of ADL, we included different types of scales: the Stroke Impact Scale (SIS-ADL), an evaluator-based measure (FIM), and the Nottingham Extended ADL (NEADL). Additionally, we included the Wolf Motor Function Test (WMFT) to test the specificity of the neural index for ADL.

According to the gating-by-inhibition model, temporal alpha is involved before responses to filter the involvement of non-motor areas, followed by a release of inhibition in the central-frontal region to enhance motor-related activity. To our knowledge, no study has examined the relationship between alpha power and clinical outcomes in stroke patients. To narrow down the exploration, we focused on three EEG indexes related to motor preparation and how these indexes relate to different hybrid tDCS stimulations and their functional roles. Given the PMC’s role in motor learning and cognition, we expect that PMC tDCS would show correlations between ADL-related measures and pre-response alpha power in the temporal area. Specifically, increased temporal alpha power for inhibiting non-motor involvement before responses, associated with PMC tDCS, should positively correlate with improvements in ADL measures. On the other hand, since the M1 is closely linked to the late stage of motor control, particularly motor execution, we anticipate that M1 tDCS would show a positive correlation between post-response alpha power in motor regions (central-frontal area) and ADL-related measures. This correlation suggests that reduced central-frontal alpha power, indicating a release from inhibition to enhance motor activity, observed after response onset is associated with post-training ADL benefits. Finally, to form a single composite index for EEG topography, we contrasted the two alpha power values (temporal and central-frontal regions) and explored its sensitivity to various ADL measures and the WMFT. To test these hypotheses, we conducted rigorous correlation analyses to identify the unique contributions of alpha power in motor production to ADL in relation to each hybrid tDCS training. The WMFT results served as a reference for the specificity of the neural indexes to ADL.

Given the distinct roles of the PMC and M1 in motor function and the gating-by-inhibition model, we anticipate that PMC tDCS would exhibit a more consistent neural-behavioral correlation than M1 tDCS. ADL performance, especially extended ADL, relies on more than simple motor movements; it also involves cognitive functions related to planning (such as doing housework and writing letters), which are closely linked to the PMC. During rehabilitation, pre-brain-damage complicated motor sequence commands that involve cognitive functions may not be helpful. Hence, proper suppression in the temporal lobe before movement initiation could prevent such potential interference, reallocating motor-related resources to the current motor command. Without controlling potential interference from the temporal lobe, direct enhancement of the motor region may not be effective, which may be the case for M1 tDCS.

## 2. Materials and Methods

### 2.1. Patient Population

**Figure 1:**
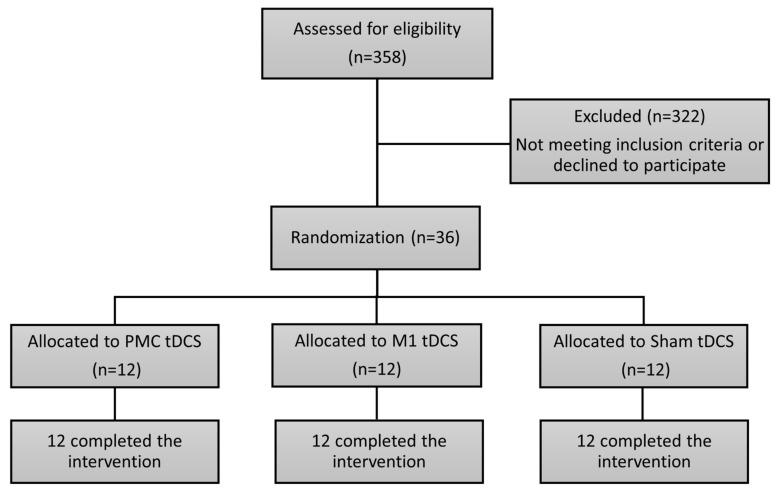
CONSORT patient flow chart.

A total of 36 eligible patients with stroke were recruited (Figure 1). They were recruited from Taipei Tzu Chi Hospital and Taipei Chang Gung Memorial Hospital in Taiwan. The inclusion criteria were: (1) an age range from 45 to 85 years; (2) Fugl-Meyer assessment (FMA) scores between 18 and 56, indicating moderate to mild impairments; (3) a documented history of unilateral stroke occurring at least 6 months prior to enrollment; (4) adequate cognitive function to comprehend and adhere to study instructions, which was measured by the Mini-Mental State Examination (≥24). Individuals were excluded from participation if they met any of the following criteria: (1) had Botulinum toxin injections in the past 3 months; (2) the upper limbs had excessive muscle tension or joint contracture; (3) concurrent presence of other neurological disorders (e.g., Parkinson’s disease, multiple sclerosis) or any mental disorders (e.g., depression); (4) a history of substance use disorder; (5) unstable cardiovascular conditions (untreated hypertension or heart failure); (6) presence of contraindications to undergoing tDCS, including epilepsy history, possession of a pacemaker, existence of metallic implants within the body, brain surgery history, etc. [24]; (7) pregnancy; (8) existence of trauma, brain tumor, or arteriovenous malformation at the site of the stimulation; and (9) ongoing involvement in other concurrent research projects.

All participants were given detailed information regarding the experimental design, research purpose, and possible risks of the study before signing the informed consent form.

### 2.2. Design and Intervention

This is a randomized, double-blinded, placebo-controlled clinical trial designed with pre-training and post-training. The study protocol is duly registered with clinical.trials.gov under the registration identifier NCT04655209. The participants were first stratified by the severity of their motor impairment (mild FMA: 41–56 to moderate FMA: 18–40) [25] and brain damage side (right vs. left cerebrovascular disease). Eligible participants in each stratum were randomly assigned to 1 of the 3 tDCS intervention groups: M1 tDCS before MT (M1 group), PMC tDCS before MT (PMC group), and sham tDCS before MT (Sham group). This assignment was conducted by a graduate student using a web-based randomization tool (freely available at http://www.randomizer.org/, accessed on 3 March 2017) [26].

The participants of the PMC group first received tDCS over the ipsilesional PMC without any active arm practice for 20 min, followed by a 20 min MT training session. The procedure for the M1 and Sham groups were identical as the PMC group, except that tDCS was applied on ipsilesional M1. All participants received the MT intervention 90 min/day, 5 days/week, for 4 consecutive weeks. A mirror was placed in participants’ sagittal planes during MT. Participants were required to look at the reflection of the non-paretic arm in the mirror, imagine it as the paretic arm and perform bilateral arm movements as simultaneously as possible. The MT training consisted of (1) intransitive movements, including distal and proximal arm/hand movements such as wrist extension/flexion, forearm pronation/supination and elbow flexion extension, and (2) transitive movements, such as placing pegs in holes or flipping a card [27]. The amount of time exposed to intransitive and transitive movements was balanced for each participant. The participants practiced 30 intransitive and 30 transitive tasks over the 20 training sessions, and the actual movements were selected based on each individual’s need. After 60 min of MT, the participants received 30 min of functional task training in those tasks opted for that were difficult for them to perform. The functional tasks involved meaningful daily activities with either hand or with both hands, such as grasping a cup with the paretic hand, wringing out water from a wet towel with both hands, or stabilizing a bowl with the paretic hand and scooping food with the non-paretic hand.

During the EEG acquisition, the participants were instructed to press the corresponding keys in response to the direction of the arrow on the screen. If the arrow pointed to the left, the participants then were asked to press the space bar. If the arrow pointed to the right, the participants were asked to press “0” on the keypad.

To date, only three studies have investigated the treatment effect of tDCS combined with MT, and only one study used a very similar stimulation protocol and collected ADL outcomes as in the current study [28]. However, this study did not correlate EEG index with ADL measures. Therefore, the sample size estimation for this study was calculated based on the effect size of the Nottingham Extended Activities of Daily Living (NEADL) ($\eta^{2}$ = 0.28) reported by Liao et al. This resulted in an estimated sample size of 11 participants per group.

### 2.3. tDCS Protocol

The tDCS was delivered by a battery-driven, direct current stimulator (StarStim, Neuroelectrics, Barcelona, Spain) through 2 saline-soaked surface sponge electrodes. The anode electrode was placed over the M1 (C3/C4 of the international 10–20 EEG electrode system) of the ipsilesional hemisphere, while the cathode electrode was placed over contralesional FP1/FP2. The anodal electrode for the PMC group was placed at the ipsilesional PMC, which was 2.5 cm anterior to C3/C4. The long axis of the anodal electrode was placed parallel to the central sulcus [20]. The electrodes were secured with Velcro bands. The size of the electrodes was 35 cm^2^, and the stimulation intensity was 2 mA, resulting in a current density of 0.057 mA/cm^2^ [29], which was well within the current safety limit [30]. For the sham tDCS, the stimulation intensity was first ramped up to 2 mA in 15 s and then ramped down to 0 within the next 30 s [19]. This method has been shown to be efficient for blinding patients [31].

The tDCS protocol (2 mA of anodal tDCS for 20 min) of the current study was developed based on following considerations. First, anodal tDCS stimulation has been shown to produce more consistent modulation effects than the cathodal tDCS [32]. Second, the anodal tDCS on the M1 and the cathodal tDCS on contralesional M1 have been applied in stroke patients with a range of 10 to 30 min of stimulation and a range of stimulation intensity between 1 to 2 mA [33,34]. Furthermore, tDCS with an intensity of 2 mA and duration of 20 min was effective at increasing cortical excitability and producing long-lasting effects [35,36]. Third, the training sessions of combined tDCS with upper limb rehabilitation were between 14 to 30 sessions [33,34], so we decided to opt for 20 sessions. Finally, one study systematically investigated the timing of applying tDCS before, during, or after a robotic arm training in patients with stroke and found improvement was pronounced when tDCS was provided before the training [37]. Thus, we decided to deliver tDCS stimulation before the MT to facilitate the treatment outcomes.

### 2.4. EEG Acquisition and Preprocessing

Continuous EEG was recorded with a cap with 32 flat actiCAP electrodes (Brain Products GmbH, Gilching, Germany) placed according to the international 10–20 system, with a sampling rate of 500 Hz. The forehead was chosen as ground and mastoids were used as the reference. Four additional electrodes were placed on the face to record horizontal and vertical eye movements. The horizontal electro-oculogram was recorded from electrodes placed at the outer canthus of each eye and the vertical electro-oculogram from electrodes placed above and below the left eye. The EEG was amplified with BrainVision LiveAmp (Brain Products GmbH, Gilching, Germany), and impedances were kept below 5 Ω.

EEG data were processed offline using EEGLAB [38] and generally followed an established preprocessing pipeline [39]. First, all the signals were digitally filtered to 0.5–40 Hz and line noise was removed using CleanLine (cleanline function in EEGLAB, Computational Neuroscience Laboratory, Salk Institute, La Jolla, CA, USA). We then focused on EEG events with contralesional responses for further analysis. Epochs were cut from −1.0 to +1.0 s relative to response initiation, which was contralateral to the lesion hemisphere. A minimum of 50 artefact-free epochs were used for further analyses in every participant. Filtered data were then re-referenced to linked mastoids. Physiological noise such as eye movements and blinks were removed through Independent Component Analysis (ICA, “pop_runica” function in EEGLAB), which resulted in an averaged removal of 2.3 components per participant.

### 2.5. Clinical Measures

Health-related quality of life was evaluated with the ADL domain of Stroke Impact Scale (SIS) 3.0. The SIS-ADL consists of 10 test items. The participants were asked to rate each item on a 5-point Likert-type scale regarding the perceived difficulty in completing the task. The SIS 3.0 has satisfactory psychometric properties [40].

The Functional Independence Measure (FIM) is one of the most common methods of assessing ADL and consists of 18 sub-items: motor items comprise 13 sub-items, and cognitive items comprise 5 sub-items [41]. Each item is scored on a 7-point ordinal scale from 1 to 7, with 1 point indicating full assistance and 7 points indicating full independence. The FIM instrument has been validated and shown to be reliable in the stroke population [42].

The Nottingham Extended Activities of Daily Living Scale (NEADL) was selected as the primary measure for evaluating instrumental ADL because it has been shown to have good psychometric properties (e.g., reliability and responsiveness) in stroke patients [43]. In addition, the NEADL does not exhibit significant floor or ceiling effects in stroke patients, and therefore it can be used across a wide range of stroke patients [44]. The NEADL consists of 22 items within four categories of daily activities, including mobility, kitchen, domestic, and leisure activities. The NEADL scale uses a 4-point rating scale. Higher scores indicate greater functional independence.

The upper extremity was assessed by the Wolf Motor Function Test (WMFT) via 15 timed function-based tasks and 2 strength-based tasks. The strength tasks involved upper extremities (raising a weight strapped on the arm to a box; hand dynamometer for grip strength. Each strength task was assessed by raters on a 6-point ordinal scale. A lower WMFT-time performance indicates faster movement, whereas a higher WMFT-strength score suggests better quality of movement. The reliability of the WMFT is excellent [45].

### 2.6. EEG Indexes

The preprocessed data were then used for EEG analysis. Time–frequency decomposition was performed through the wavelet transform method conducting on overlapping windows, each with a range from −1 to 1 s relative to response onset. The pad ratio was set to 4. This procedure was repeated on each channel individually. The yield complex-valued results were first doubled for all positive frequencies, and alpha power was computed as the squared amplitude in the 8–12 Hz frequency range. The alpha frequency range was chosen based on past relevant studies [12]. Next, we computed absolute alpha power without baseline correction by applying median-scaled log transformation [10∙log10] to each participant.

We first inspected the power spectrum plot for alpha power reduction at the central-frontal area and an alpha surge at temporal sites around the onset of response. To solve the issue of the multiple comparison problem, the time windows used to quantify the alpha power at central-frontal and temporal sites was first explored through a nonparametric statistical test [46]. This method first repeatedly drew subsets from a combined data set and calculated test statistics from this random partition. The *p*-value was generated based on the histogram, the proportion of random partitions, to conclude if the experimental conditions were significantly different. This testing could help identify meaningful clusters of time windows for later stages of parametric statistical analyses. Two time windows of clusters were identified with significance: −65–0 and 0–127 ms; the alpha power values within these two windows were targeted for further statistical analyses. Central-frontal alpha power was averaged across frontal (F3, Fz, F4) and central (C3, CP1, C4, CP2) sites, while temporal alpha power was averaged from bilateral temporal (T7, F7, CP5, T8, F8, CP6) sites. To focus on the feature of topography, we also tried to combine the previous two measures into a single composite: temporal alpha divided by central-frontal alpha. Thus, a higher value indicates better psychomotor efficiency, as suggested by the gating-by-inhibition model.

### 2.7. Statistical Analysis

The Kolmogorov–Smirnov test and Levene’s test were applied to test for normality and homogeneity of variance on clinical variables. If the normality assumption was violated, between-group comparison was performed using the Kruskal–Wallis Test, and a within-group comparison was performed using the Friedman test. For normally distributed data, two-way mixed model analysis of variance (ANOVA) with factors of Groups (PMC, M1, Sham) and Training (pre- vs. post-training) was used to examine the effects of intervention on WMFT, ADL, and relevant EEG indexes. If the interaction of the ANOVA survived, follow-up post-hoc comparisons with the Bonferroni correction within each measurement were carried out accordingly. To establish robust associations between behavioral and EEG measurements, we used skipped correlations [47,48] to avoid issues with Pearson correlation [49]. The skipped correlation has been proposed to prevent distortion by the outliers, which made a small sample size study highly susceptible. The skipped-correlation first estimated multivariate location of the data scatter by minimum covariance determinant [50], while rejecting outliers with using the box-plot rule [51]. The remaining data were then used to compute Pearson correlations and their associated *t*-values. Similar to parametric analysis, a higher value of the *t* statistic means the greater evidence against the null hypothesis. Finally, to further reduce heteroscedasticity (dependence between variables), a percentile bootstrap 95% critical interval (2.5 and 97.5 percentiles) was performed for each correlation analysis and served as a determinant for rejection of the null hypothesis. A significance was accepted if 0 was not within the CI range. All the above statistical tests and the regression model were conducted by MATLAB (The MathWorks, Natick, MA, USA) and the Robust Correlation toolbox [47].

## 3. Results

### 3.1. Demographical Features

As shown in Table 1, there were no significant differences in baseline demographic characteristics (*p* > 0.05 for all), indicating that the three tDCS groups were well matched on all relevant demographic attributes.

### 3.2. ADL Outcomes: SIS-ADL, FIM, NEADL

Separate 2-way mixed-ANOVAs were conducted on SIS-ADL, FIM, and NEADL to assess the impact of the hybrid tDCS training (Table 2). In SIS-ADL, the interaction of Time*Group was not significant [*F*(2,33) < 1, *p* = 0.48], suggesting that training-related improvement was similar across groups. The main effects of Time [*F*(2,33) < 1, *p* = 0.67] and Group [*F*(1,33) < 1, *p* = 0.44] were also not significant. In the FIM score, interaction [*F*(2,33) = 1.38, *p* = 0.27] and main effects were not significant [both *F*s < 1, both *p*s > 0.29]. Similarly, in NEADL, all the interaction [*F*(2,33) < 1, *p* = 0.85] and main effects were not significant [both *F*s < 1, both *p*s > 0.38]. It appears that the influence of tDCS was not evident in ADL outcomes.

### 3.3. Motor Ability Outcome: WMFT-Time, WMFT-Strength

Similarly, 2-way mixed-ANOVAs were conducted on two subscales of WMFT. In the time-based tasks, neither the interaction [*F*(2,33) < 1, *p* = 0.58] nor the main effects were significant [both *F*s < 1.23, both *p*s > 0.31]. Clearly, the training-induced effects were identical (Table 2). For the strength-based tasks, although the interaction was not significant [*F*(2,33) < 1, *p* = 0.70], the main effect of time was significant [*F*(1,33) = 26.37, *p* < 0.01], which suggests a general post-training improvement across the three groups (Table 2). Follow-up post-hoc comparison revealed this improvement was mainly attributed to the M1 tDCS group [*t*(11) = 4.62, *p* < 0.01, corrected]. The difference between the three groups was significant [*F*(1,33) = 1.37, *p* = 0.27].

### 3.4. Alpha Power

**Figure 2:**
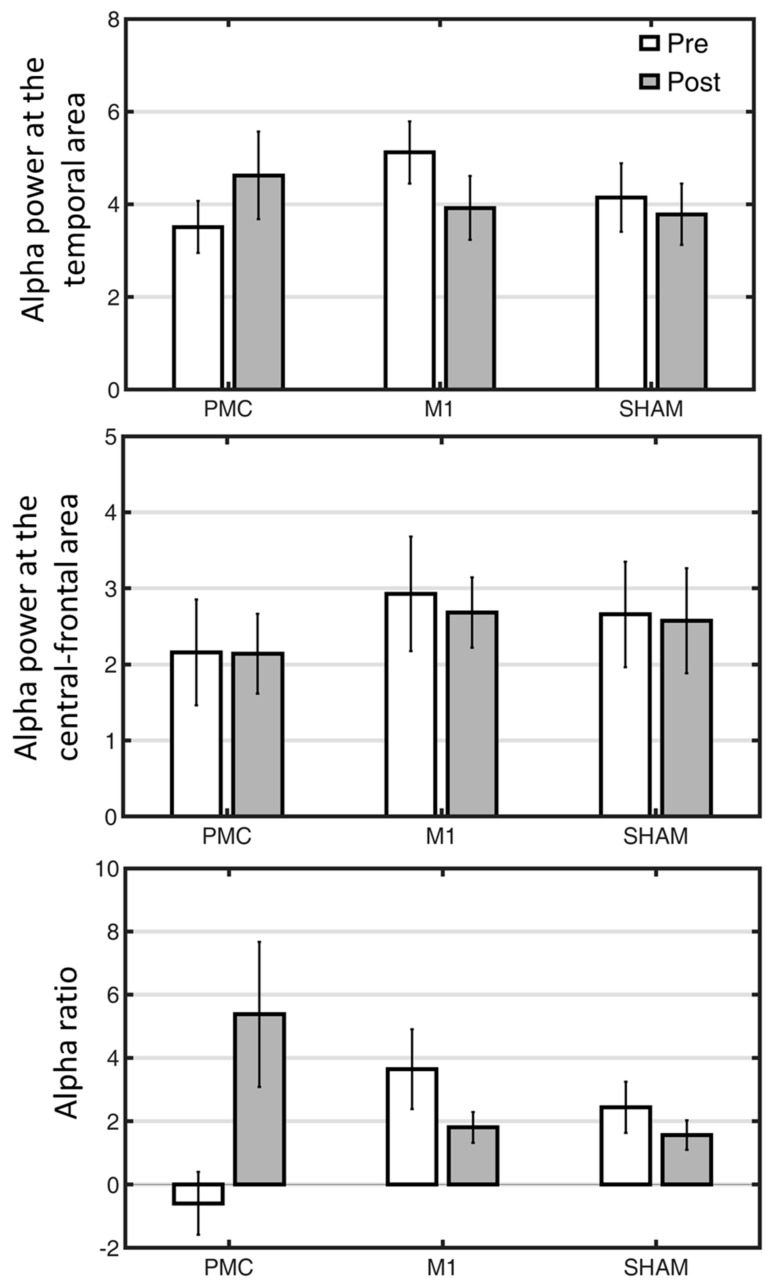
The changes of three EEG indexes as a function of tDCS interventions (PMC, M1, Sham tDCS groups) and assessment time points (pre vs. post). PMC = premotor cortex, M1 = primary motor cortex. Error bars denote standard error of mean.

Regarding the exploration of the overall sensitivity of the EEG indexes in response to different tDCS stimulations (Figure 1), the examination of the sensitivity focused on alpha power at temporal, central-frontal areas and a composite of alpha ratios computed by the previous two measures. A separate 2-way mixed ANOVA was conducted to investigate its responsiveness to the three tDCS trainings. For the index of alpha power in the temporal lobe, the insignificant interaction [*F*(2,33) = 1.34, *p* = 0.27] suggested that the changes in alpha power among the three groups were comparable, although the PMC tDCS group and the other two groups showed opposite numerical patterns (Figure 2). The main effects of Time and Group were both insignificant [both *F*s < 1, *p*s > 0.72]. For alpha power obtained from the central-frontal region, the interaction and main effects were all not significant [all *F*s < 1, *p*s > 0.72]. Unlike the previous measures, the interaction for the alpha ratio index was significant [*F*(2,33) = 4.13, *p* = 0.03], suggesting that the effects of the three tDCS interventions on the alpha ratio were dissimilar. Follow-up post hoc did not find any significant differences, although there was a numerical trend of post-training increase in the PMC tDCS group [*t*(11) = 1.86, *p* = 0.09] but not in other two groups [both *p*s > 0.22]. Together, these suggest that the alpha power may have the potential to differentiate distinct tDCS interventions and could provide more information than behavioral measures. To further link these EEG indexes to various ADLs in relation to different tDCS stimulations, we performed exploratory correlation analyses.

### 3.5. Neural-Behavioral Correlation: SIS-ADL

**Figure 3:**
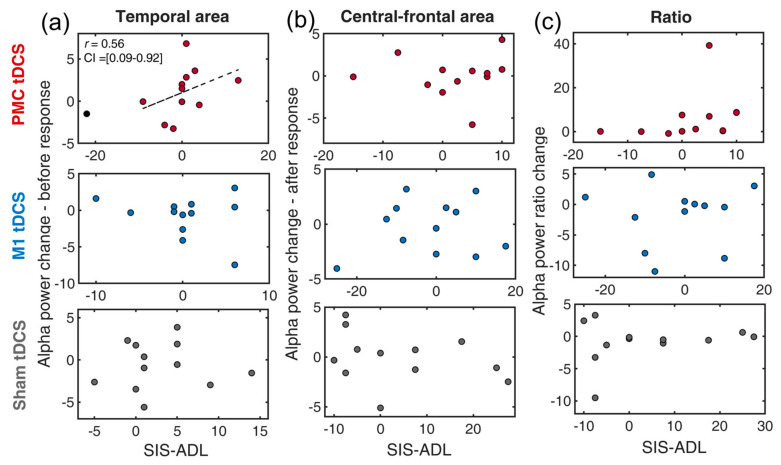
Scatter plots of the changes of EEG indexes and SIS-ADL for a combination of three tDCS group (premotor tDCS, M1 tDCS, Sham tDCS) and different alpha power indexes: (**a**) temporal alpha, (**b**) central-frontal alpha, (**c**) alpha ratio. The dotted line indicates the best linear fit of the data after removing outliers. The circles in black denote outliers. PMC = premotor cortex, M1 = primary motor cortex. SIS-ADL = Stroke Impact Scale-activity of daily living.

The performance differences between pre- and post-training on SIS-ADL and alpha power in the temporal, central-frontal regions were first computed to conduct correlation analyses for the neural-behavioral correlation. The skipped correlation analysis was conducted for each tDCS group and EEG indexes separately (Figure 3). In the temporal region associated with cortical inhibition, a positive correlation was found between temporal alpha and SIS-ADL in the PMC tDCS group. This indicated that, after training, the stronger inhibition found in the temporal lobe was associated with greater gains in SIS-ADL [*r* = 0.56, *t* = 2.15, CI = 0.09, 0.92]. In contrast, this correlation was absent in the M1 tDCS group [*r* = 0.39, *t* = 1.33] or the Sham tDCS group [*r* = −0.40, *t* = −1.37].

For alpha power in the central-frontal region, none of the correlation was significant [all *t*s < 1.04]. Similarly, no significant results were found for the index of alpha ratios (contrasting alpha power at temporal and central-frontal regions) [all *t*s < 1.97].

### 3.6. Neural-Behavioral Correlation: FIM

**Figure 4:**
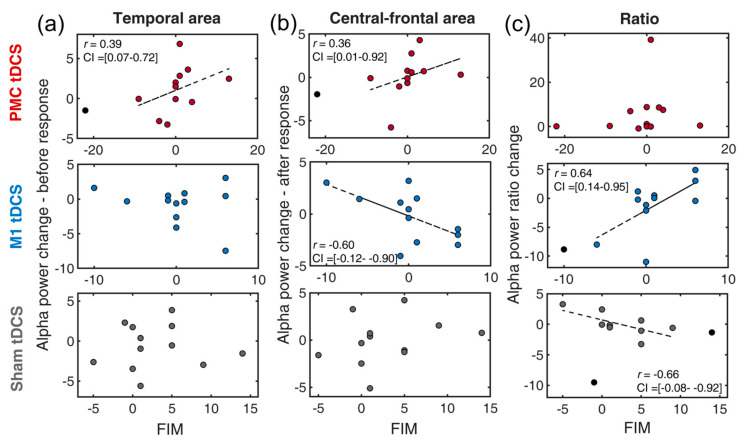
Scatter plots of the changes of EEG indexes and FIM for a combination of three tDCS group (premotor tDCS, M1 tDCS, Sham tDCS) and different alpha power indexes: (**a**) temporal alpha, (**b**) central-frontal alpha, (**c**) alpha ratio. PMC = premotor cortex, M1 = primary motor cortex. FIM = Functional Independence Measure. The circles in black denote outliers. The dotted line indicates the best linear fit of the data after removing outliers.

FIM measures the degree of disability for an individual to carry out activities of daily living with assistance. Similarly, domain scores of FIM and alpha power values (post-pre) were first generated prior to the skipped correlation analyses. In the temporal region (Figure 4a), increased alpha power was only found to correlate with enhanced FIM in the PMC tDCS group [*r* = 0.39, *t* = 1.33, CI = 0.07, 0.72] but not in the other two tDCS groups [all *t*s < 1]. This suggests that increased temporal cortical inhibition, potentially induced by PMC tDCS, facilitated improvement in FIM.

In the central-frontal region, alpha powers were found to have a positive correlation in the PMC tDCS group [*r* = 0.36, *t* = 1.21, CI = 0.01, 0.92] but a negative correlation in the M1 tDCS group [*r* = −0.60, *t* = −2.38, CI = −0.12, −0.90], respectively (Figure 3b). The negative correlation of the M1 tDCS group is in line with our expectation that less inhibition after a response over the motor region would benefit motor generation. The finding of the positive correlation in the PMC tDCS is not part of our expectation. This rather suggests that less central-frontal inhibition (higher alpha power) would benefit FIM recovery. Lastly, the composite of alpha ratios revealed a positive association in the M1 tDCS group [*r* = 0.64, *t* = 2.60, CI = 0.14, 0.95] and a negative association in the Sham group [*r* = −0.66, *t* = −2.80, CI = −0.08, −0.92]. This clearly implies that M1 tDCS might assist FIM improvement but did the opposite in the Sham (MT alone) tDCS.

### 3.7. Neural-Behavioral Correlation: NEADL

**Figure 5:**
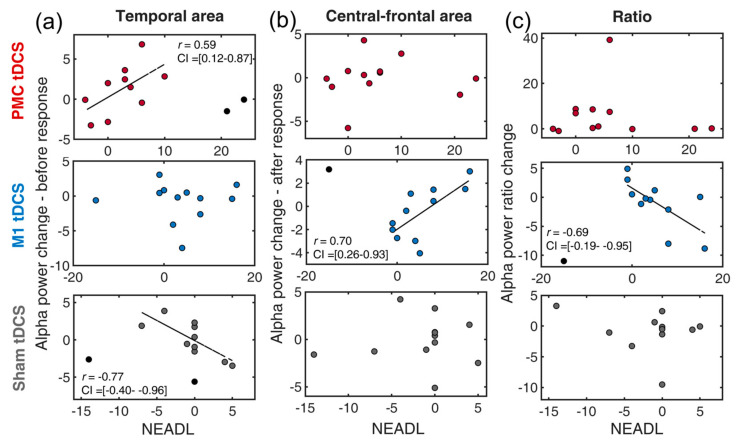
Scatter plots of the changes of EEG indexes and NEADL for a combination of three tDCS groups (premotor tDCS, M1 tDCS, Sham tDCS) and different alpha power indexes: (**a**) temporal alpha, (**b**) central-frontal alpha, (**c**) alpha ratio. PMC = premotor cortex, M1 = primary motor cortex. NEADL = Nottingham Extended Activities of Daily Living Scale. The circles in black denote outliers. The dotted line indicates the best linear fit of the data after removing outliers.

Compared to the previous two ADL measures, NEADL provides extended, comprehensive measures of disability in community life. Difference scores were first computed (post–pre) for all relevant measures. The skipped-correlation revealed a positive correlation between temporal alpha (Figure 5a) and the NEADL in the PMC tDCS group [*r* = 0.59, *t* = 2.29, CI = 0.12, 0.87] but no significant correlation in the M1 tDCS group [*r* = −0.06, *t* < 1] and a negative correlation in the Sham tDCS [*r* = −0.77, *t* = −3.86, CI = −0.40, −0.96] group. This finding demonstrated that PMC tDCS-associated temporal suppression was also beneficial for extended ADL, which is in line with our hypothesis.

In the central-frontal region (Figure 4b), the M1 tDCS group exhibited a negative correlation [*r* = 0.70, *t* = 3.08, CI = 0.26, 0.93], whereas the other two groups showed no significant correlation [both *t*s < 1.72]. These results imply that more central-frontal activity induced by the M1 tDCS stimulation did not contribute to the NEADL. For the alpha composite measure, surprisingly, the M1 tDCS group also found a negative correlation [*r* = −0.69, *t* = −3.01, CI = −0.19, −0.95]. This suggests that higher psychomotor efficiency induced by M1 tDCS was associated with decreased NEADL performance. The correlation was not seen in the PMC tDCS and the Sham tDCS groups [both *t*s < 1.20].

### 3.8. Neural-Behavioral Correlation: WMFT

The correlation analyses conducted here aimed to ensure the earlier observed correlations were specific to ADL. For WMFT-Time, the correlation analyses conducted on the combination of tDCS interventions and EEG indexes revealed no significant correlations [all *t*s < 1.10]. Similarly, for WMFT-Strength, none of relationships was significant [all *t*s < 1.08]. As anticipated, EEG indexes were not responsive to motor-related measures.

## 4. Discussion

The current study adopted novel theory-based EEG indexes to examine the impact of three hybrid tDCS on different aspects of patients’ recovery. We first used different alpha power indexes and various clinical measures to assess the influence of the combined tDCS. Post-ADL improvement among the three tDCS interventions was not detected; however, one of the alpha indexes differentiated, suggesting that EEG measures might detect additional information. To further connect the functional roles of the EEG indexes to ADL in relation to the PMC and M1 roles in the motor circuit, we correlated individual clinical performance with the three EEG indexes derived from the model. The results clearly demonstrated that ADL improvements, including SIS-ADL, FIM, and NEADL, reliably varied with alpha power in the temporal lobe exclusively in the PMC tDCS group. Moreover, from the same group, more central-frontal alpha power was also found to correlate with ADL, which indicates that applying tDCS over the PMC caused less motor activity and was beneficial for ADL. In contrast, alternation of alpha power in the central-frontal region was found to vary with ADL in the M1 tDCS group, although its benefit depends on the complexity of the ADL. More motor activity (indicated by less alpha at the central-frontal region) is associated with improved basic ADL (FIM), while less motor activity (indicated by higher alpha in the central-frontal region) is associated with improved extended ADL (NEADL). Additionally, for the index of alpha ratios, worse psychomotor efficiency (lower alpha ratio) was related to improved ADL. None of the EEG indexes mentioned was found to associate with ADL measure in the Sham group. Finally, the motor ability measure (WMFT) did not relate to any neural index in any of the hybrid tDCS groups, which supports the notion that the proposed EEG indexes have the potentials to selectively track ADL. The relevant mechanism for each tDCS effect was discussed in the following sections.

In our results from patients with chronic stroke, enhancement of ADL was not seen across various measures, which may seem inconsistent with previous findings [5,6,7]. tDCS alone has been suggested to be effective in improving ADL [7]. It has also been found to be beneficial when combined with other intervention techniques, but mainly in acute stroke populations [5,6]. Our sample consisted entirely of chronic stroke patients, which may explain our subtle behavioral ADL results. Additionally, past studies combined different therapies, such as physiotherapy and occupational therapy [5]. Another explanation could be the various outcome measures of ADL used in other studies. In a review focusing on the influence of tDCS on ADL [7], common measures included the Barthel ADL Index, the Rivermead ADL Assessment, and the Modified Rankin Scale, which were not included in our study. Together with variations in the tDCS protocols, these factors may explain our delicate ADL effects.

We provided the following explanations for the correlation results identified earlier. The correlation analyses were performed to reflect the associations between EEG indexes and distinct aspects of recovery, ranging from physical to ADL functions. While SIS-ADL, FIM and NEADL all assess the activity of daily life, the main difference is that FIM tends to address relatively basic daily activities, whereas NEADL focuses on extended activities within a broader living context (instrumental activities of daily living). From the three tDCS interventions, PMC tDCS consistently contribute to improvement in scores in the majority measures (Figure 3a, Figure 4a and Figure 5a) except WMFT (served as control purpose). Moreover, its involvement mainly arose from a heightened alpha power in the temporal lobe, which is according to the gating-by-inhibition model that it should filter irrelevant non-motor interference. Such a pattern was not seen in other two tDCS groups, which again proves its close link to the role of the PMC. In the central-frontal area, however, more alpha power was correlated with an improving FIM score, suggesting that PMC tDCS induced less activity in the motor area and was beneficial to FIM improvement, while the lesser activity was only confined to after the response onset. This is still partially consistent with our prediction that the PMC is mainly involved in the earlier stage of motor preparation relating to suppressing non-motor interference. Overall, these findings support the view that, compared to the M1, the PMC is not directly involved in motor production but rather focuses on motor planning [52]. Moreover, one study also suggests that the PMC is a hub for motor and cognitive networks, which may serve as another potential explanation for the relationship between temporal alpha power effects and ADL [53].

The correlation results found in the M1 tDCS group (Figure 4b and Figure 5b) are explained as follows. It is expected that a selective correlation exists between central-frontal (motor area) alpha of the M1 tDCS and clinical measures for its role of direct involvement in motor production. An anticipated negative correlation was found in FIM, which suggests less alpha power (less inhibition) during motor execution was beneficial for basic daily life activity. In contrast, the opposite correlation was found in NEADL, which indicates that an increased suppression of motor activity promoted extended daily life activity. These contrasting findings imply that the improvement of ADL may depend on its content and its relative activity in the M1. In other words, the enhancement in basic ADL might rely on sustained M1 activity, whereas extended ADL requires less M1 activity. In the NEADL, many items (e.g., leisure, domestic activities) require more than strength or mobility but more concerns the ability to plan or generate a strategy for coordinating individual motor movements for a task goal that involves several sub-tasks [54]. A similar notion can be applied to the index of alpha ratios, as an expected pattern was selectively found in FIM, but an opposite pattern was seen in NEADL. Together, this emphasizes the notion that the M1 tDCS seems to be more related to motor activity and the types of ADL.

In the Sham tDCS group, negative correlations were observed between alpha ratios and the FIM and between temporal alpha and the NEADL. Although we did not explicitly make predictions for the Sham group based on the gating-by-inhibition model, the findings appeared to be inconsistent with the model’s predictions. This could indicate either the insensitivity of the EEG indexes for mirror therapy or the superiority of the hybrid tDCS approach. A review paper summarized alpha, beta, and mu rhythms as commonly associated neural indexes with MT [55], which included alpha rhythm and served as a gating mechanism in the gating-by-inhibition model. Thus, we are more inclined to believe that our correlation results reflect the robustness of hybrid tDCS in assisting ADL recovery in post-stroke patients.

The finding that temporal and central-frontal alpha correlated with ADL-related measures but not with WMFT clearly demonstrates the specificity in ADL. This aligns with our hypothesis that PMC tDCS would consistently show ADL improvements and hints at the link between the gating-by-inhibition model, particularly the suppression of temporal activity, and ADL-related performance. Moreover, this specificity led us to suspect two potential underlying therapeutic networks, each serving the PMC and M1 tDCS interventions. For PMC tDCS, the inhibition of temporal activity consistently contributed to both basic and extended ADL measures, suggesting the same mechanism of temporal inhibition involved in different ADL measures. For M1 tDCS, its mechanism was closely linked to the central-frontal region, where stronger activity is associated with simple ADL and lower activity with extended ADL.

We adopted the gating-by-inhibition model to explain the clinical impact of hybrid tDCS on ADL improvement. First, the EEG indexes in alpha oscillatory activity derived from the model correlated with several ADL measures. The role of alpha oscillatory is to suppress irrelevant temporal activity while promoting motor activity through a release of inhibition in motor activity. We further showed that central-frontal motor activity modulated by central-frontal alpha could selectively relate to either basic or extended ADL when applying M1 tDCS. This evidence suggests that different hybrid tDCS approaches exert their influence through distinct routes, reflected in different levels of clinical measures: no effect in function-based task, M1 tDCS associated with basic ADL, and PMC tDCS associated with extended ADL. The clinical implication is that the therapists are able to select a specific hybrid tDCS approach depending on the treatment goal set for the stroke patients. For example, if the main goal for the stroke patient is to improve basic ADL such as dressing, a M1 tDCS hybrid approach would be a plausible way to achieve the goal.

The application of the gating-by-inhibition model on ADL enhancement is reminiscent of other proposed mechanisms underlying the effects of MT and tDCS on ADL, particularly the aspect of reducing competing cortical activity to achieve a more balanced mode for motor execution. One example is the rebalanced interhemispheric activity account, which is an accepted explanation for MT. Michielsen and colleagues [56] used fMRI to identify changes in the activation balance within the primary motor cortex of the affected hemisphere in the MT intervention group, finding that MT shifted activation toward the affected lesion, inducing more symmetrical activity between the two hemispheres. Another example relates to the application of tDCS in ADL. A meta-analysis found that cathodal tDCS benefits ADL by downregulating the overactive non-affected brain hemisphere, restoring the balance of excitatory and inhibitory interactions between both hemispheres [57]. This study highlights the importance of downregulating overactive non-lesioned brain regions to optimize tDCS protocols. Overall, these two accounts, as well as the gating-by-inhibition model, emphasize the need to suppress competing cortical activity for a balance state in motor recovery. The main difference is that our account further considers the competing activity in the temporal lobe.

Our study may be limited by the small sample size within each group, making it susceptible to individual variability and reducing statistical sensitivity. Another limitation could be the concomitant use of certain medications for treatment of other health issues, such as antihypertensive drugs, which might be associated with reducing cognitive dysfunction. Finally, the generalization of the EEG indexes in ADL is limited, as we only examined the neural-behavioral correlations in the context of the combination of tDCS and MT intervention.

## 5. Conclusions

The current study was designed to examine the influences of the three hybrid tDCS interventions on a patient’s motor function and activity of daily living and the associated EEG indexes that were rooted from the gating-by-inhibition model. EEG indexes seemed to be more sensitive than the behavioral data, as evident by dissimilar EEG results (alpha ratio) between the three groups but a comparable ADL improvement. To strengthen the association between the EEG markers and the type of the tDCS intervention, we correlated the ADL improvement and the changes of the neural indexes. The correlation results clearly demonstrated that alpha power at the temporal lobe, which relates to inhibiting cortical activity in non-motor regions, was consistently linked to the changes of the ADL and exclusively observed when applying PMC tDCS intervention. On the other hand, alpha power at the central-frontal region, which relates a relaxation of cortical inhibition, also connected to ADL and was mainly observed in the M1 tDCS group. However, the ADL enhancement was dependent on the level of the alpha power: more central-frontal alpha power could be beneficial to basic ADL but less was helpful for extended ADL. These correlation findings together enlighten the functional roles of the premotor and primary motor cortex in the novel tDCS intervention in conjunction of MT. Alpha power in the temporal lobe appeared to be a core component in facilitating ADL recovery in hybrid PMC tDCS. In conclusion, this research demonstrated the additive benefits of introducing theory-driven neural indexes in explaining ADL.

## Figures and Tables

**Table 1:** Demographic and clinical characteristics of the participants.

Variables	PMC (n = 12)	M1 (n = 12)	Sham (n = 12)	*p*-Value
Age (year)	58.95 (12.40)	54.33 (14.60)	64.03 (7.25)	0.22
Gender/Male	8 (66.67%)	11 (91.67%)	10 (83.33%)	0.29 ^a^
Education (year)	13.75 (2.01)	13.25 (4.81)	11.67 (3.28)	0.12
Lesion/left hemisphere (%)	6 (50%)	6 (50%)	6 (50%)	1.00
Onset time (months)	53.92 (39.79)	48.00 (42.44)	26.33 (16.85)	0.24
MMSE	32.42 (10.61)	33.08 (11.88)	34.5 (11.29)	0.84
FMA	28.33 (1.56)	28.42 (1.73)	28.67 (1.15)	0.94

Values are mean (standard deviation) or n (%). Categorial variables were tested with Chi-square test (*p*-value with ^a^) while continuous variables were tested by Kruskal–Wallis test (for non-normalized data). PMC = premotor group, M1 = primary motor group, Sham = sham group, MMSE = Mini-Mental State Examination, FMA = Fugl-Meyer assessment.

**Table 2:** Clinical performances for the three tDCS groups.

	PMC (n = 12)		M1 (n = 12)		Sham (n = 12)	
Variables	Pre	Post	Pre	Post	Pre	Post
SIS-ADL	74.58 (14.88)	76.46 (13.79)	71.25 (17.21)	69.72 (19.47)	68.96 (17.24)	72.92 (13.97)
FIM	113.58 (9.48)	112.33 (12.78)	106.50 (9.47)	106.67 (12.22)	106.50 (10.32)	109.42 (9.39)
NEADL	36.92 (15.63)	42.75 (13.05)	33.67 (13.35)	37.33 (13.41)	34.25 (13.07)	32.83 (12.22)
WMFT-Time	11.04 (6.48)	12.28 (7.42)	14.25 (5.22)	10.75 (4.82)	9.72 (5.37)	10.56 (6.50)
WMFT-Strength	2.76 (0.67)	2.91 (0.58)	2.51 (0.61)	2.69 (0.65)	3.06 (0.80)	3.23 (0.83)

Values are mean (standard deviation). PMC = premotor group, M1 = primary motor group, Sham = sham group. Pre = pre-training. Post = post-training. SIS-ADL = Stroke Impact Scale-Activities of Daily Living. FIM = Functional Independence Measure. NEADL = Nottingham Extended Activities of Daily Living scale. WMFT = Wolf Motor Function Test.

## Data Availability

The raw data supporting the conclusions of this article will be made available by the authors on request.
